# Foreign Body in the Trachea

**Published:** 1842-07-16

**Authors:** 


*Foreign body in the Trachea.*—M. Guersant also showed a child who had been brought to the hospital fourteen days after having swallowed a haricot bean, which passed into the trachea; the symptoms of suffocation occurred at intervals only, and the child was submitted to medical treatment for a few days by some of M. Guersant's colleagues, who did not think that there was any foreign body in the air-passages. The operation was not performed until the eighteenth day after the accident. As soon as the trachea was opened the haricot appeared at the incision, and was immediately extracted. The child recovered perfectly.—*Provincial Medical Journal.* April 16, 1842.

